# Editorial

**Published:** 1844-09-07

**Authors:** 


### THE MEDICAL EXAMINER.

PHILADELPHIA, SEPT. 7, 1844.

### HEALTH OF PHILADELPHIA.

In a former number we spoke of the remarkable exemption from disease experienced by the inhabitants of Philadelphia of latter years, and especially the present season. We have some reason for believing that a careful comparison of the number of deaths with that of the population, for a number of years past, will show that the mortality has been regularly diminishing. We have not time at present to enter on the investigation of this matter to a sufficient extent to settle the point, but as it is one of great interest, we shall be glad if some of our friends, whose attention is directed in that way, will furnish us with a paper on the subject, or the data which will enable us to make out such a statement. We have before us at this moment the official "*statements of deaths, with the diseases and ages*, in the City and Liberties of Philadelphia," during the years 1842 and 1843.

In some parts of the world, fevers are regarded as the great outlets of human existence; in Philadelphia, diseases of the respiratory organs seem to constitute the great sources of mortality.

The total number of deaths in 1842 was 5943; in 1843, 5526; being four hundred and seventeen less in 1843 than in 1842, notwithstanding the increase of population within that period.

| | In 1842. | In 1843. |
|---|---|---|
| The deaths by consumption were | 774 | 743 |
| " inflammation of the lungs, | 293 | 248 |
| " " of the bronchi, | 155 | 155 |
| " scarlet fever, - - | 220 | 395 |
| " cholera infantum, - | 235 | 268 |
| " still-born, - - | 385 | 371 |
| " convulsions, - - | 331 | 280 |
| " old age, - - | 145 | 154 |
| " small-pox, - - | 156 | 36 |
| " measles, - - | 24 | 1 |
| " congestive fever, - | 10 | 10 |

It must strike any one as remarkable that, in so large a population, there should have been but *one* death from *measles*, in 1843; and only 25 in two years! To those who have imbibed the belief that consumption does not occur in natives of Philadelphia, it will be incomprehensible how so many die of that disease, constituting, after deducting the deaths from old age, accidents, and the still-born, something like one-fourth of the whole number.

### HYDROPATHY.

One of the worshippers at the shrine of this new folly, writing from Gräfenberg to W. L. Garrison, says, Priessnitz is "about 40 years old—a little over perhaps—a peasant, who probably never in all his life read a book on medical science. He has no book learning," &c. A wise man, that Priessnitz, no doubt!

According to this excellent judge: "when the names of Hippocrates, Galen, and all who figure in the medical profession—*the art of poisoning, according to rule*—have passed into oblivion, the name of Priessnitz will be cherished by millions as a benefactor of his race!" We suppose this will not happen until the names of Hippocrates, Galen, and all the rest, *have* "passed into oblivion!" But, in the meanwhile, what is to become of Homœopathy?

### UNIVERSITY OF KING'S COLLEGE.

This is the title of an institution established by the British Government, at the City of Toronto, for the promotion of learning in Canada, and endowed, as we understand, with extensive privileges.

Recently, a Medical Faculty has been organized, consisting of the following gentlemen:

H. Sullivan, M. R. C. S. L. Practical Anatomy.
W. C. Gwynne, M. B. Anatomy and Physiology.
H. H. Croft, Esq. Chemistry.
J. King, M. D. Theory and Practice of Medicine.
W. Beaumont, M.R.C.S.L. Principles and Practice of Surgery.
G. Herrick, M. D. Midwifery and Diseases of Women and Children.
W. B. Nicol, Esq. Materia Medica and Pharmacy.

The following are the qualifications requisite for admission to the final examination:

1st. Having attained the age of twenty-one years.

2d. Having passed five years in the acquisition of Medical knowledge, three of which must have been occupied in attendance on Medical Lectures; two in Schools, recognized by the University; and one, at least, in the Medical School of this University.

3d. Having attended the following courses of Lectures and Hospital Practice:

| | |
|---|---|
| Anatomy and Physiology,<br>Theory and Practice of Medicine,<br>Principles and Practice of Surgery,<br>Practical Anatomy, with Dissections | 2 of 6 months. |
| Chemistry,<br>Midwifery and Diseases of Women and Children,<br>Materia Medica and Pharmacy, | 1 of 6 months. |
| Practical Chemistry, | 1 of 3 months. |

With eighteen months' attendance on a Medical and Surgical Hospital, containing not less than eighty beds—twelve months of which must be during the winter, at an Institution where Clinical Lectures are delivered on Medicine and Surgery.

### MEDICAL EXAMINATIONS AND LICENSES IN CUBA.

We copy the following letter from the Boston Medical and Surgical Journal of the 21st ult. It contains some curious matter that may interest our readers generally, but we extract it more especially on account of the younger members of the profession who may be inclined to seek employment in any of the Spanish possessions, in all of which, we have reason to believe, similar regulations exist. Of the authenticity of the information, there can be no doubt. Beside the respectable channel through which it comes, we have seen a letter from an eminent American physician, now residing in Havana, which confirms every particular.

[The following interesting letter was addressed to Professor W. Parker, of New York, by a former pupil of his, practising medicine in Havana, and has been politely forwarded to us by Prof. P. As it was not apparently written for publication, we withhold the name of the writer.]

*Havana, April* 25, 1844.

"Dear Sir,—Thinking that it may be of some interest to you to know the course of the medical examination to which foreign physicians are subjected before they can legally practise on this island, I have thought that I would give you the particulars of the late formalities and examinations through which I have passed at the Royal University of this city.

In the first place, I would say, that in commencing this undertaking, the candidate should provide himself with an almost inexhaustible stock of PATIENCE; for the annoyances, formalities and vexatious delays with which he will meet, will very soon drain anything like a moderate supply of that most necessary and useful attribute of mind. I arrived here from Trinidad on the 7th of March, and it was not until the 12th of April that with all my efforts I could get a day named for my examination. It is true that the occurrence of the *Semana Santa*, or Holy Week, and a number of other holydays, occasioned a loss of time that perhaps might not have otherwise taken place. But holydays or no holydays, there will be detention and constant delay; poco à poco, manana, manana—little by little, to-morrow, and to-morrow, is, I may say, the unvarying course of Spanish tribunals. Many a valuable "to-morrow" and "day after to-morrow" comes and goes, and to your grief and cost do you find that your business is no nearer to a conclusion than before, but remains quite *in statu quo*.

The first thing to be done, if the individual has not already done it, is to take out a *carta de domicilio*, giving him authority to remain in the island and establish himself where he may choose in the practice of his profession. This requires about three days, and costs, if one understands the language and can manage the matter for himself, four dollars and a half; if he is obliged to hire another to do it for him, it will cost him a couple more. Next he must take a petition in due form to the President of the Subinspection of Studies, who is the Captain General himself, praying that his Excellency will deign to give the necessary order to the end that he may be legally qualified for the exercise of his profession upon this island. This petition must be handed to the Secretary of the Subinspection of Studies, and be laid before the Junta or Medical Council, before it can be acted upon. This grave body meets but once a week, on Wednesday evening, and I suppose no earthly consideration would make them meet any oftener. The *carta de domicilio* and the diploma of the candidate, must be also laid before the Junta, the latter being previously certified by the American Consul, which costs two dollars. The Junta meet, and the papers being laid before them in due form, they order that the candidate proceed to take the necessary steps for the attainment of his object. After, perhaps, a couple of weeks, and sometimes double and treble that number, the candidate is ordered to prove his identity in the office of the Secretary of the Subinspection of Studies. That is, to prove by three good witnesses that he is the individual spoken of in the diploma, and no other; to prove, as a gentleman who went as witness with me facetiously remarked, *que usted es usted*—that *you are you.* Your witnesses must be natives of your own country, American citizens, known and established in this city, and must have been acquainted with you at least for some considerable time. They are examined separately, and a clerk formally takes down the deposition of each one in writing. After all this ceremony is finished, the Secretary of the Subinspection of Studies writes an official letter to the Rector of the University, stating that having gone through all the necessary formalities in his office, he now sends you to undergo the requisite examinations at the Royal University. And here begins somewhat deeply *la funcion del dinero*, the play of money, which is anything but amusing to the person that has it to pay. The *derochos* or fees at this office are twenty dollars. I paid my money, took the official despatch, and repaired immediately to the University, where I delivered it in the Secretary's office, and by insisting somewhat I at last obtained the appointment of the next day, the 13th of April, at 1 o'clock, P. M., for my first examination. The Rector appoints three examiners from among the professors of the University, and the Subinspection of Studies sends one as a delegate from their body. On the day of the first examination, previous to that act, I had to make the following heavy deposites, viz: one hundred and twenty-five dollars paid into the Treasury of the University, and one hundred and twenty-five dollars paid into the hands of the Beadle, to be distributed by him among the examiners, for both of which sums receipts were given me, which I had to deliver to the Secretary, before entering the hall of examination.

At last the hour arrived, and my cane being duly taken from me at the door by one of the porters, and duly placed in the corner with the other gold-headed, wise-looking, doctorial canes, I was ushered by the Beadle into the august presence of my dignified examiners. The Beadle is an exceedingly important and busy personage on these occasions; he is master of ceremonies, ushers you in and out, and sits by your side during the whole of the examination, and is in fact a sort of body-guard or constable to see that you do not infringe any of those sacred rules of etiquette and formality, in the observance of which the Spaniards have ever been so excessively punctilious. None but a black dress, I was duly informed beforehand by the illustrious Beadle, would be considered *de etiqueta;* so I took care to go diplomatically arrayed—*vestido de negro* from head to foot. The room for examination is a large and very stately one, hung with crimson, and at the end opposite the door of entrance is the stage or pulpit for the Rector, over which hangs a portrait of the young Queen of Spain. A row of permanent arm-chairs for the examiners extends from each side of the pulpit towards the door, and at the end of these, between the two rows, is a table and seat covered with red cloth, and upon this the poor wight of a candidate is placed, as a fair target to be shot at from both sides, without even a back or a side to his seat, or a single object to conceal his bashfulness or mortification, should some unlucky missile but too sorely wound him.

I had been seated but a few moments when the Reverend Rector, attired in full canonicals, black surplice and gown, lace cuffs and collar, entered and took his chair of state. My watchful guardian, the Beadle, ordered me to rise as he passed, and on my attempting, without further honors, to sit down again, he told me not to sit down until his lordship, *Su Usia*, was seated. When the very Reverend Senior was comfortably composed in his seat, the Dean of the Faculty, Dr. C. V. rang a little bell and called for *los espedientes*, that is, the different papers showing that I had duly taken all the legal steps and formalities *conforme a lo despuerto*, to arrive at the surely not enviable position I was then occupying. Probably the most important of these *espedientes*, were the receipts showing that the two hundred and fifty dollars were safe in the coffers of the University, and in the important hands of the punctilious Beadle. I confess that at the first examination all this formality, dignity, cremony and etiquette, quite surprised and confounded. Unlike, also, our strictly private examinations, these are free to the public, and a number of the Students of the University were present, the door of the hall being wide open. The Beadle, too, lost no occasion of scrupulously demonstrating the importance of his functions; in the course of the examination, noticing that I used, in replying to the professors, the word *usted*, you—the usual very respectful mode of address among Spaniards, he whispered to me, in a low voice, and told me that I must there use the word *usia*, your honor, your lordship. I felt at that moment little inclined to use compliments with anybody. The noise, too, from the street, through the open door, was sometimes almost deafening, and I seated at such a formal distance from my examiners that I several times could not hear their questions at all. However, the hour terminated, as all hours will, but to me it was an excessively long and disagreeable one. It tends, also, not a little to increase your discomfort, to know that the hundred and twenty-five dollars paid to the examiners will be *entirely lost*, in case they reject you; for if they give you another trial some months after, which they sometimes do, *their* fee has to be paid over again in full amount, as anything less than that sum is not considered a compensation for the privilege of being screwed!

The matters touched upon in the examination were of much the same nature as at our colleges, but they were discussed with much more length and minuteness. They examine on everything pertaining to medicine and surgery, except chemistry—anatomy and physiology, theory and practice of medicine, clinical medicine and surgery, materia medica, surgical diseases and operations, obstetrics, and all matters, which are various, that come under the head of medical jurisprudence. Dr. C. V. is the professor of medical jurisprudence, and one of the most accomplished and intelligent professors of the University. I would cheerfully add, too, that he was decidedly the most affable, fair and considerate of my examiners, and this has generally been the experience of all the candidates when he has been one of the Board. A kind look, and a candid, assuring manner in the professor examining, has a wonderful effect to soothe and animate the timid, agitated pupil. This pleasant manner Dr. C. V. most eminently possesses, which cannot be said by any means of all the rest.

The next trial was appointed for the day but one after, Monday, the 15th inst., at half past 4 o'clock, P. M., at the Hospital de San Juan de Dios, where, at the appointed hour, I met the professors, and after examining the medical case which they gave me, we all adjourned to the University, and I there passed another *examine* of nearly an hour. The diagnosis of the case which they gave me was very easy, for it was a young man covered from head to foot with the smallpox, some of the pustules being now in the drying stage. They examined me minutely on the nature and treatment of this disease, and on contagion and infection in general, and took another wide ramble over all the branches of medicine and surgery.

The third and last examination was appointed for the following A. M., at 7 o'clock, to meet the professors again at St. John's Hospital, and be given a surgical case to examine and be examined upon; and also, should there be a recent subject, to perform some operations upon the dead body. There was no *cadaver* that morning, which circumstance I did not at all regret. They showed me a boy with congenital hare-lip, and a case of fracture of the patella, and after examining these and walking around the Hospital, we went again to the University, where I had a minute examination of thirty-five minutes upon the cases I had seen, and upon a variety of other subjects besides. In the second and third examinations, having recovered my confidence and become somewhat accustomed to their formal mode of procedure, I succeeded much better than in the first.

The last examination being concluded, I was ushered out by Monsieur le Beadle, the door was shut, and the professors went into conclave. In about three minutes the Beadle came out and informed me that I was approved—*usted esta aprobado*, which were indeed cheering words after all the harassing formalities and delays, and the ordeal of three rigid examinations, through which I had passed. At the conclusion, I was required to take an oath of fidelity to the Queen of Spain, and obedience to the Spanish laws while I remain upon the island. This was read to me by the Dean of the Faculty, Dr. C. V., standing upon the stage, and here again el Senor Beadle, untiring in his zeal for forms, motioned to me to fall upon my knees while the oath was being read, but the worthy Dean, with great consideration, interrupted the important official, and told me it was not necessary.

On receiving the diploma or *titulo* which they give, you have to pay another twenty dollars into the Treasury of the University; making, with the certification of your diploma, two hundred and ninety-two dollars. In the course of the proceedings you will have to use two or three sheets of stamped paper at half a dollar a sheet, and you will find also, that, notwithstanding the enormous sums which you have paid to the *high* functionaries, the lazy porters at the door will be most vexatiously teazing you for a fee—*alguna cosa para refrescar*. Another dollar to them, together with the letter of domiciliation, will make the whole expenses amount to very near three hundred dollars. Understanding the Spanish language, I did not have to employ an interpreter; if I had been obliged to do so, it would have been an additional expense of fifty dollars, and that is what an American physician now practicing here informed me he had to pay.

A few years since, the examinations were a mere form, almost dispensed with, the payment of five hundred dollars being by far the most important and essential formality. On this old regime I have been informed that numbers of persons quite unqualified, bought licenses—apothecaries, barber-surgeons, &c. About two years since the fees were reduced (?) to their present rates, and the examinations commenced in good earnest, and in the month of March just passed, the new regulations concerning physicians and surgeons, and all matters relating to them, have been published in a pamphlet form, of which every physician and surgeon is required to have a copy. Perhaps it would be interesting to you to know its title, and one or two of its more important articles. It is called "Reglamento de Medicina y Ciruja Formado por la Subinspecion de Estudios de las Islas de Cuba y Puerto Rico y aprobado por Su Magested en Real Orden de tres de Enero de 1844." Chapter IV., article 14, says, "No persons can exercise in the Islands of Cuba and Puerto Rico the profession of Medicine and Surgery, nor the branches of Dentist, Bleeder and Midwife, without having the corresponding title given to them by the competent authority." Article 15—"Those who, without a legal title, shall exercise any branch of the healing art, or shall exceed the faculties which their title concedes to them, shall be fined, the competent summary information having been previously given by the local judge, in the sum of one hundred dollars for the first offence, and in default of payment one month's imprisonment; for the second offence, two hundred dollars or two month's imprisonment; and for the third offence, three hundred dollars or three month's imprisonment; with the right, besides, in either of the three cases, in the event of any disastrous result from the illegal practice, to prosecute according to law for the purpose of condign punishment." Article 17 says—"In the same manner foreign physicians must present to the Subinspection of Studies their respective titles, legalized in due form, and prove also the identity of their persons; but this tribunal can in no case license them to practice, except there take place, before the Board of the respective faculty, the proof examinations and practical exercises prescribed in the 100th article, of the general Plan of Studies, and the deposite which is spoken of in the 123d article of the Regulations of the University." So you see that quacks have no chance of success *here*, and the door is narrow and the way difficult and expensive for even regular practitioners of any nation whatever.

I close with something of the same advice as that with which I commenced, to any professional brother about to undertake this arduous enterprise: "Go doubly armed with *patience and money*, for both the one and the other will meet with heavy draughts; and go well prepared for the examination, for a rigid and severe one you may be sure of receiving."

